# Using Temporal Features to Provide Data-Driven Clinical Early Warnings for Chronic Obstructive Pulmonary Disease and Asthma Care Management: Protocol for a Secondary Analysis

**DOI:** 10.2196/13783

**Published:** 2019-06-06

**Authors:** Gang Luo, Bryan L Stone, Corinna Koebnick, Shan He, David H Au, Xiaoming Sheng, Maureen A Murtaugh, Katherine A Sward, Michael Schatz, Robert S Zeiger, Giana H Davidson, Flory L Nkoy

**Affiliations:** 1 Department of Biomedical Informatics and Medical Education University of Washington Seattle, WA United States; 2 Department of Pediatrics University of Utah Salt Lake City, UT United States; 3 Department of Research & Evaluation Kaiser Permanente Southern California Pasadena, CA United States; 4 Care Transformation Intermountain Healthcare Salt Lake City, UT United States; 5 Center of Innovation for Veteran-Centered & Value-Driven Care VA Puget Sound Health Care System Seattle, WA United States; 6 Division of Pulmonary and Critical Care Medicine, Department of Medicine University of Washington Seattle, WA United States; 7 College of Nursing University of Utah Salt Lake City, UT United States; 8 Department of Family and Preventive Medicine University of Utah Salt Lake City, UT United States; 9 Department of Allergy Kaiser Permanente Southern California San Diego, CA United States; 10 Department of Surgery University of Washington Seattle, WA United States

**Keywords:** decision support techniques, forecasting, machine learning, patient care management

## Abstract

**Background:**

Both chronic obstructive pulmonary disease (COPD) and asthma incur heavy health care burdens. To support tailored preventive care for these 2 diseases, predictive modeling is widely used to give warnings and to identify patients for care management. However, 3 gaps exist in current modeling methods owing to rarely factoring in temporal aspects showing trends and early health change: (1) existing models seldom use temporal features and often give late warnings, making care reactive. A health risk is often found at a relatively late stage of declining health, when the risk of a poor outcome is high and resolving the issue is difficult and costly. A typical model predicts patient outcomes in the next 12 months. This often does not warn early enough. If a patient will actually be hospitalized for COPD next week, intervening now could be too late to avoid the hospitalization. If temporal features were used, this patient could potentially be identified a few weeks earlier to institute preventive therapy; (2) existing models often miss many temporal features with high predictive power and have low accuracy. This makes care management enroll many patients not needing it and overlook over half of the patients needing it the most; (3) existing models often give no information on why a patient is at high risk nor about possible interventions to mitigate risk, causing busy care managers to spend more time reviewing charts and to miss suited interventions. Typical automatic explanation methods cannot handle longitudinal attributes and fully address these issues.

**Objective:**

To fill these gaps so that more COPD and asthma patients will receive more appropriate and timely care, we will develop comprehensible data-driven methods to provide accurate early warnings of poor outcomes and to suggest tailored interventions, making care more proactive, efficient, and effective.

**Methods:**

By conducting a secondary data analysis and surveys, the study will: (1) use temporal features to provide accurate early warnings of poor outcomes and assess the potential impact on prediction accuracy, risk warning timeliness, and outcomes; (2) automatically identify actionable temporal risk factors for each patient at high risk for future hospital use and assess the impact on prediction accuracy and outcomes; and (3) assess the impact of actionable information on clinicians’ acceptance of early warnings and on perceived care plan quality.

**Results:**

We are obtaining clinical and administrative datasets from 3 leading health care systems’ enterprise data warehouses. We plan to start data analysis in 2020 and finish our study in 2025.

**Conclusions:**

Techniques to be developed in this study can boost risk warning timeliness, model accuracy, and generalizability; improve patient finding for preventive care; help form tailored care plans; advance machine learning for many clinical applications; and be generalized for many other chronic diseases.

**International Registered Report Identifier (IRRID):**

PRR1-10.2196/13783

## Introduction

### Three Major Gaps in the Current Predictive Modeling Method for Implementing Care Management

COPD and asthma are chronic respiratory diseases incurring heavy health care burdens on society, health care systems, and payers. In the United States, COPD affects over 6.5% of adults, is the third leading cause of death, and incurs 700,000 inpatient stays, 1.5 million emergency room visits, and US $32 billion in cost annually [1]. Asthma affects 8% of adults [2] and 9.6% of children [3,4] and incurs 3630 deaths, 493,000 inpatient stays, 1.8 million emergency room visits, and US $56 billion in cost annually [2,5]. As a service intended to prevent poor outcomes such as hospitalization, care management is widely adopted to provide tailored preventive care for COPD and asthma patients. Purchased by most large employers and offered by almost all private health plans [6-8], care management is a collaborative process to evaluate, plan, implement, coordinate, and monitor options and services to accommodate an individual’s health and service needs [9]. In care management, a care manager calls a patient regularly, helps arrange for medical appointments, and schedules health and related services. Appropriate use of care management can cut hospital use (emergency room visits and inpatient stays) by up to 40% [7,10-15], lower cost by up to 15% [11-16], and enhance patient adherence, quality of life, and satisfaction by 30% to 60% [10].

Predictive models are widely used, for example, by health plans in 9 of 12 regions [17], as the best method [18] to warn of poor outcomes and to identify COPD and asthma patients for care management [6-8]. Multiple models have been built for predicting the health outcomes of individual COPD and asthma patients [19-28]. However*,* current modeling methods have 3 major gaps restricting their effectiveness owing to inadequate use of temporal features showing trends and early health change. A temporal feature, such as the slope of pulmonary function across the last year, is an independent variable formed by transforming longitudinal attributes.

#### Gap 1: Late Warning

Existing models for predicting the health outcomes of individual COPD and asthma patients seldom use temporal features [19-28] and often give late warnings, making care reactive and missing opportunities for clinical and therapy teams to intervene early to reduce the risk of poor outcomes. A health risk is often identified at a relatively late stage of declining health, when the chance of a poor outcome is high and resolving the issue is difficult and costly. A typical model predicts patient outcomes in the next, say, 12 months. For patients with imminent poor outcomes, this does not warn early enough. If a patient will actually be hospitalized for COPD next week, intervening now could be too late to avoid hospitalization. If temporal features were used, this patient could be identified a few weeks or months earlier; when health decline is still at an early stage, resolving the issue is easier and preventing hospitalization is likely.

#### Gap 2: Low Prediction Accuracy

Models for predicting a patient’s health outcome and cost typically have low accuracy. When projecting the health outcome of a patient, the accuracy measure of area under the receiver operating characteristic curve (AUC) is typically much lower than 0.8 [19-28]. When projecting the health care cost of a patient, the accuracy measure of *R*^2^ is typically lower than 25% [29,30], and the mean error is as big as the mean cost [31]. These large errors in prediction results create difficulty in properly aligning care management’s use with the patients needing it the most [10].

Care management can require over US $5000 per person per year [11] and usually enrolls only 1% to 3% of patients because of resource limits [32]. For patients predicted to have the worst outcomes or the largest costs [10,33], care managers review patient charts and manually make allocation decisions.

A small percentage of patients use most of the health care resources and costs. The upper 20% of patients use 80% of the resources and costs. The upper 1% use 25% [19,32,34]. Accurately identifying patients at high risk for poor outcomes or large health care costs is critical for effective targeted application of care management resources. Yet, Weir et al [33] showed that in the upper 10% of patients who actually spent the largest health care costs, over 60% of them were not included in the upper 10% risk group identified by a predictive model. In the upper 1% of patients who actually spent the largest health care costs, around 50% and over 80% of them were not included in the identified upper 10% and 1% risk groups, respectively. Assume the care management program could take 1% of all patients. In this case, even if the care managers could afford to examine the upper 10% risk group found by the predictive model and manually make correct decisions for enrollment, the care managers would still not find around half of the upper 1% of patients who spent the largest health care costs. For COPD and asthma, if we could identify 10% more of the upper 1% of patients who spent the largest health care costs and enroll them in care management, we could boost outcomes and spare possibly up to US $120 million in COPD care [1] and US $210 million in asthma care each year [19-21]. In general, owing to the large patient population, a small boost in accuracy will benefit numerous patients and have a big positive impact.

Current models for predicting the health outcomes and health care costs of individual COPD and asthma patients have low prediction accuracy for several reasons:

1. Many temporal features with high predictive power are either frequently unused in an existing model or yet to be found. Google recently applied long short-term memory (LSTM) [35], one kind of a deep neural network, to all the attributes in the electronic health record to automatically learn temporal features from longitudinal data [36]. For forecasting each of the 3 outcomes: long hospital stay, unanticipated readmissions within 30 days, and in-hospital mortality, this increased the AUC by approximately 10% [36]. Multiple other studies [37-39] showed similar results for a variety of clinical prediction tasks. This aligns with what has taken place in areas such as video classification, natural language processing, and speech recognition, where temporal features that LSTM automatically learned from data outperform those mined from data by other methods or specified by experts [40,41].
2. Although >40 risk factors for undesirable outcomes in COPD/asthma have been identified [19,20,23,28,42-48], an existing model usually uses only a few (eg, <10) [19-24,26-28]. Existing models were often constructed based on data obtained from clinical trials or old fashioned electronic health records collecting limited variables [49]. No published model adopts all of the known risk factors available in modern electronic health records collecting extensive variables [49].
3. Environmental information such as air quality and weather variables are known to impact COPD and asthma outcomes [43,50-52], but with rare exceptions [25], are infrequently used in existing models.

#### Gap 3: Lack of Information on Why Patients Are at High Risk for Poor Outcomes and Possible Interventions to Mitigate Risk

Before enrolling a patient, care managers need to know why the patient is at high risk for a poor outcome and about possible interventions to mitigate risk. Complex predictive models, which include most machine learning models such as LSTM, give no explanatory or prescriptive information. Frequently, a patient’s records have many variables on hundreds of pages accumulated over a long period of time [53]. Unlike physicians who see patients from time to time, care managers often have not previously seen the records when needing to make enrollment decisions. When the model offers no explanation, busy care managers often spend extra time reviewing the records to find the reasons. This is time consuming and difficult.

A care manager may use subjective, variable judgment to form a care plan, but may miss some suited interventions because of 2 factors:

1. Several reasons can make a patient at high risk for a poor outcome. Each reason is shown by a feature combination as a risk pattern. For instance, the ratio of inhaled steroid to beta agonist dispensing to the patient decreased over 12 months and the sulfur dioxide level was ≥3 parts per million for ≥5 days in the past week. Many features exist. Like any human, an ordinary care manager can deal with ≤9 information items simultaneously [54], making it difficult to identify all reasons from numerous possible feature combinations.
2. Huge variation in practice, often by 1.6 to 5.6 times, appears across care managers, facilities, and regions [34,55-58].

Missing suited interventions can degrade outcomes. Typical automatic explanation methods [59,60] do not handle longitudinal attributes and cannot fully address these issues.

#### Our Proposed Solutions

To fill the gaps for more COPD and asthma patients to receive appropriate and timely care, we will (1) use temporal features to provide accurate early warnings of poor outcomes and assess the potential impact on prediction accuracy, risk warning timeliness, and outcomes; (2) automatically identify actionable temporal risk factors for each patient at high risk for future hospital use and assess the impact on prediction accuracy and outcomes; (3) assess actionable information’s impact on clinicians’ acceptance of early warnings and on perceived care plan quality. Here, actionable information refers to the explanations and their linked interventions provided by our automated approach.

### Innovation

This study will lead to several innovations. We will develop new, general informatics techniques. We will transform care management for COPD and asthma by directing it to the patients needing it in a more timely fashion and more precisely than current methods:

1. We will build models to predict a patient’s hospital use earlier and more accurately than current models, which often give late warnings and have low accuracy.
2. We will be the first to semiautomatically extract predictive and clinically meaningful temporal features from longitudinal medical data. This process helps us address data quality issues and automatically find and drop uninformative variables. All of these boost model accuracy and generalizability and reduce the effort needed to build models usable in clinical practice. Currently, to build such models, clinicians typically need to manually identify such features, which is difficult and time consuming.
3. We will be the first to provide rule-based automatic explanations of machine learning prediction results directly on longitudinal data. Explanations are critical for care managers to understand the results to make appropriate care management enrollment and intervention decisions. Compared with other forms of automatic explanations such as that used in Rajkomar et al [36], rule-based explanations are easier to understand and can more directly suggest actionable interventions. Most automatic explanation methods [60], including our previous one [59], for machine learning prediction results cannot handle longitudinal attributes. Also, our previous method [59] gives explanations for a limited portion of patients. We will improve our previous method, handle longitudinal attributes, and expand automatic explanations’ coverage of patients.
4. We will be the first to automatically identify actionable temporal risk factors and suggest interventions based on inclusion of objective data. Currently, care managers use subjective, variable judgment to manually form care plans. Some suited interventions for patients at high risk for a poor outcome get missed. Also, care managers provide a finite input on the patient to the other clinical care team members. With automatic explanations and suggested interventions in hand, care managers can pass this tailored information to the other clinical care team members so they can act accordingly. This could transform the care management process and make it more effective via closer collaboration between care managers and the other clinical care team members.
5. Current models for predicting the health outcomes of individual COPD and asthma patients were built mostly using a small number of patients (eg, <1000) or variables (eg, <10) [19-28], making it difficult to identify many predictive features and the interactions among them. Air quality and weather variables impact COPD and asthma outcomes [43,50-52] but are rarely used in existing models. The predictive power of many known risk factors for undesirable outcomes is unused. Also, many predictive features have not yet been found. In contrast, we will use many patients and variables, enabling us to identify more predictive features and the interactions among them. The variables will include air quality, weather, and patient variables, cover many known risk factors for undesirable outcomes, and be used to find new predictive features in a data-driven way. Many features are new, capturing trends that existing models rarely touch.

To build and validate models for predicting the health outcomes of individual COPD and asthma patients, we will use data from 4 different electronic health record systems HELP, HELP2, Cerner, and Epic. This boosts model generalizability. In contrast, every existing model for predicting the health outcomes of individual COPD and asthma patients was built using data from only 1 electronic health record system [19-28].

In short, this study is significant as it will produce new techniques to advance machine learning for clinical applications and potentially transform preventive care for more patients to receive appropriate and timely care. The wide use of these techniques could boost outcomes and save resources.

## Methods

### Computing Environment

All experiments will be done on a secure computer cluster at the University of Washington Medicine (UWM) that is encrypted and password protected. With proper authorization, all of the UWM care manager and physician test participants and research team members can log into this computer cluster from their UWM computers. We will install Oracle database, R, Weka [61], and TensorFlow [62] to be used in the study on the computer cluster. Weka is a major open-source machine learning toolkit. It incorporates many popular machine learning algorithms including both base and ensemble algorithms, feature selection techniques, and methods for dealing with imbalanced classes [63]. TensorFlow is Google’s open-source deep neural network package.

### Datasets

We will employ clinical and administrative data from the enterprise data warehouses (EDWs) of 3 leading health care systems: Intermountain Healthcare (IH), Kaiser Permanente Southern California (KPSC), and UWM, as well as publicly available air quality and weather data. All of the data to be used are structured. We will use all patients’ data that are needed for computing health care system features [64,65], rather than only COPD and asthma patients’ data. As the largest health care system in Utah, IH has 185 clinics and 22 hospitals. The EDW of IH contains numerous variables [66]. In this study, we will start with using the following of these variables: “admission date and time; age; orders (medications, labs, exams, immunizations, imaging, and counseling), including order name, ordering provider, performing date, and result date; allergies; barriers (hearing, language, learning disability, mental status, religion, and vision); cause of death; chief complaint; death date; diagnoses; discharge date; exam result; facility seen for the patient visit; gender; health insurance; healthcare cost; height; home address; immunizations; lab test result; languages spoken; medication refills; primary care physician as listed in the electronic medical record; problem list; procedure date; procedures; provider involved in the visit; race/ethnicity; referrals; religion; visit type (inpatient, outpatient, urgent care, or emergency department); vital signs; weight” [65]. An IH data analyst will download a de-identified IH dataset, encrypt it, and transfer it to the secure computer cluster. The IH dataset has information on clinical encounters in the previous 14 years (2005 to 2018). For the previous 5 years, the IH data for adults cover over 5,786,414 clinical encounters and 878,448 adult patients (aged ≥18 years) per year. The IH data for children cover over 1,557,713 clinical encounters and 360,698 pediatric patients (aged <18 years) per year. COPD prevalence is approximately 4.1% in the IH adult population. Asthma prevalence is approximately 8.6% and 7.6% in the IH adult and pediatric population, respectively. The IH dataset provides the electronic record of care for approximately 60% of adults and approximately 95% of children in Utah [56,67]. IH devotes many resources to maintain data integrity and accuracy. Owing to its huge size and variable richness, the dataset provides many advantages for us to explore the proposed predictive models.

KPSC and UWM have similar strengths. KPSC is the largest integrated health care system in Southern California, providing care to approximately 16% of residents in 227 clinics and 15 hospitals [68]. A KPSC data analyst will download a de-identified KPSC dataset, encrypt it, and transfer it to the secure computer cluster. The KPSC dataset has information on clinical encounters in the previous 10 years (2009 to 2018). For the previous 5 years, the KPSC data for adults cover over 9,448,987 clinical encounters and 2,890,027 adult patients per year. The KPSC data for children cover more than 1,380,900 clinical encounters and 975,249 pediatric patients per year. COPD prevalence is approximately 4.1% in the KPSC adult population. Asthma prevalence is approximately 10.8% and 10.9% in the KPSC adult and pediatric population, respectively.

As the largest academic health care system in Washington, UWM has 12 clinics and 4 hospitals. A UWM data analyst will download a de-identified UWM dataset, encrypt it, and transfer it to the secure computer cluster. The UWM dataset has information on adult patient encounters in the previous 7 years (2012 to 2018). The UWM data cover over 1,714,196 clinical encounters and 277,289 adult patients per year. COPD prevalence among patients is approximately 4.1%. Asthma prevalence is approximately 9%.

In addition to the clinical and administrative data, we will use 11 air quality and weather variables, which were recorded over the previous 14 years (2005 to 2018) by the monitoring stations in the regions served by UWM, IH, and KPSC and are available from federal data sources [69,70]. These variables include ozone, sulfur dioxide, particulate matter up to 10 μm in size, particulate matter up to 2.5 μm in size, nitrogen dioxide, temperature, carbon monoxide, wind speed, relative humidity, precipitation, and dew point.

In the following, we sketch our techniques. Our design paper [71] describes the ideas in more detail. In this study, for each technique, we will flesh out its technical details, do computer coding, tune its parameters, and test it. The discussion below focuses on COPD. Whenever we mention COPD, the same applies to asthma.

### Aim 1: Use Temporal Features to Provide Accurate Early Warnings of Poor Outcomes and Assess the Impact on Prediction Accuracy.

We will semiautomatically extract predictive and clinically meaningful temporal features from patient, air quality, and weather data, and build models to predict a patient’s health outcome. Each feature involves one or more raw variables. The number of possible features is almost infinite. In addition, factors such as environmental variables beyond air quality and weather can influence patient outcomes. This study does not intend to exhaust all of the possible features and factors that can influence patient outcomes and achieve the highest possible prediction accuracy in theory. Rather, our purpose is to show that using temporal features can improve risk warning timeliness, prediction accuracy, and care management. A nontrivial boost in health outcomes can greatly benefit society. As is adequate for our target decision support application and typical with predictive modeling, our study focuses on associations.

#### Data Preprocessing

We will write Oracle database SQL queries and R and Java programs for data preprocessing. Our source code will be made freely available on a project website hosted by UWM. In our future publications on this study’s results, we will describe all of the decisions made for data preprocessing, such as the thresholds used for determining the physiologically impossible and invalid values of an attribute. We will transform all of the datasets into the Observational Medical Outcomes Partnership (OMOP) common data model format [72] and its related standardized terminologies [73]. We will extend the data model to include patient, air quality, and weather variables that are in our datasets but not covered by the original data model. We will adopt conventional techniques such as imputation to manage missing values and to find, rectify, or drop invalid values [74,75]. To avoid using too many longitudinal attributes, we will employ grouper models such as the Diagnostic Cost Group system to merge diseases, drugs, and procedures [31,34]. We will use the method given in our paper [71] to select the most relevant laboratory tests.

We will use patient, air quality, and weather variables. The patient variables include standard variables such as diagnoses that the clinical predictive modeling literature [34,55,74] has studied and many known risk factors for undesirable COPD outcomes listed in Bahadori et al [45]. For air quality and weather variables, we will do spatial interpolation [76] to obtain their daily average values at the patient’s home address from those at regional monitoring stations [77].

#### Chronic Obstructive Pulmonary Disease and Asthma Cases and Outcomes

As test cases, we will develop and test our approach using (1) COPD, (2) pediatric asthma, and (3) adult asthma. For COPD, we will adjust the criteria used by the Centers for Medicare and Medicaid Services and National Quality Forum [78-80] to incorporate outpatient and emergency room visit data [81] to find COPD patients. A patient is deemed to have COPD if he/she is ≥40 years and has one of the following:

1. 1 outpatient visit diagnosis code of COPD (International Classification of Diseases, Ninth Revision [ICD-9]: 491.21, 491.22, 491.8, 491.9, 492.8, 493.20, 493.21, 493.22, 496; International Classification of Diseases, Tenth Revision [ICD-10]: J41.8, J42, J43.*, J44.*) and ≥1 prescription of tiotropium within 6 months of the outpatient visit.
2. ≥2 outpatient or ≥1 emergency room visit diagnosis codes of COPD (ICD-9: 491.21, 491.22, 491.8, 491.9, 492.8, 493.20, 493.21, 493.22, 496; ICD-10: J41.8, J42, J43.*, J44.*).
3. ≥1 hospital primary discharge diagnosis code of COPD (ICD-9: 491.21, 491.22, 491.8, 491.9, 492.8, 493.20, 493.21, 493.22, 496; ICD-10: J41.8, J42, J43.*, J44.*).
4. ≥1 hospitalization with a primary discharge diagnosis code of respiratory failure (ICD-9: 518.81, 518.82, 518.84, 799.1; ICD-10: J80, J96.0*, J96.2*, J96.9*, R09.2) and a secondary discharge diagnosis code of acute exacerbation of COPD (ICD-9: 491.21, 491.22, 493.21, 493.22; ICD-10: J44.0, J44.1).

The outcome measure is whether a patient used the hospital (inpatient stay and emergency room visit) with a primary diagnosis of COPD (ICD-9: 491.21, 491.22, 491.8, 491.9, 492.8, 493.20, 493.21, 493.22, 496; ICD-10: J41.8, J42, J43.*, J44.*) in the subsequent year.

For asthma, we will use Schatz et al’s [20,82,83] method to find asthma patients. A patient is deemed to “have asthma if he/she has 1) ≥1 diagnosis code of asthma (ICD-9 493.*; ICD-10 J45/J46.*) or 2) ≥2 asthma-related medication dispensing records (excluding oral steroids) in a one-year period, including β-agonists (excluding oral terbutaline), inhaled steroids, other inhaled anti-inflammatory drugs, and oral leukotriene modifiers.” [84] The outcome measure is whether a patient used the hospital with a primary diagnosis of asthma (ICD-9 493.*; ICD-10 J45/J46.*) in the subsequent year.

#### Temporal Feature Extraction

We will use a new method to semiautomatically extract predictive and clinically meaningful temporal features from longitudinal data. These features will be used to build the final predictive model and to automatically identify actionable temporal risk factors for each patient at high risk for future hospital use. Our new method is semiautomatic, as its final step involves a human to extract features through visualization [71]. It generalizes to many clinical applications and is sketched as follows, with more details described in our design paper [71].

Our method uses LSTM [35], a type of deep neural network that models long-range dependencies and often reaches higher prediction accuracy than other algorithms [40]. A lot of work has been performed using LSTM to construct predictive models on medical data [36-39,85]. LSTM performs computations on a sequence of input vectors from the same patient, one vector after another. Every input vector is marked by a time step *t*. After finishing the whole sequence, LSTM gains results that are combined with static attributes such as gender [86] to predict the outcome of the patient. Every input vector contains information of one patient visit such as vital signs and diagnoses. The sequence length can differ across patients. This helps increase model accuracy, because LSTM can use as much of each patient’s information as possible, without dropping information to make every patient have the same length of history. In addition, this enables us to make timely predictions on new patients without waiting until every patient acquires history of a certain length. With information from only one visit, LSTM can start making projections on the patient.

**Figure 1:**
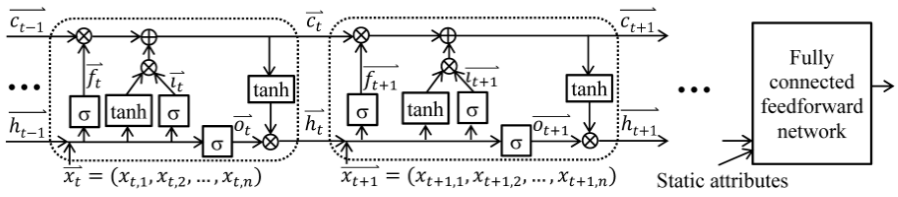
A long short-term memory network.

As Figure 1 shows, an LSTM network includes a sequence of units, one for each time step. In the figure, each unit is denoted by a rounded rectangle. ⨂ represents the element-wise multiplication. ⨁ represents the element-wise sum. A unit contains an input gate *i_t_*, a hidden state *h_t_*, an output gate *o_t_*, a forget gate *f_t_*, and a memory cell *c_t_*. The memory cell maintains long-term memory and keeps summary information from all of the previous inputs. Every element of the memory cell vector represents some learned temporal feature. As shown by Karpathy et al [87], only approximately 10% of the memory cell vector elements could be interpreted [88]. This is because LSTM puts no limit on the number of input vector elements that can connect to every memory cell vector element. All of the input vector elements could be adopted in every element of the input and forget gates’ activation vectors and connect to every memory cell vector element. Consequently, no limit is put on the number of attributes utilized in every learned temporal feature.

**Figure 2:**
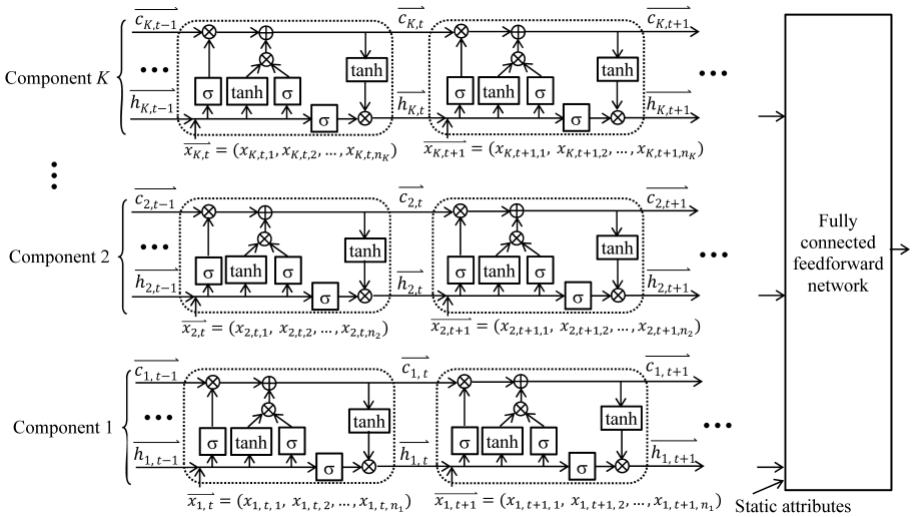
A multi-component long short-term memory network having K components.

It is difficult to understand a feature that involves many attributes. To address this issue, we will use multi-component LSTM (MCLSTM), a new type of LSTM that can automatically drop uninformative attributes. As Figure 2 shows, an MCLSTM has several component LSTM networks, each using some rather than all of the longitudinal attributes. By limiting the number of attributes connecting to every memory cell vector element, more memory cell vector elements will depict clinically meaningful and more generalizable temporal features. As LSTM often produces more accurate models than other algorithms [36-39], the learned features tend to be predictive. As patient attributes are collected at a different frequency from air quality and weather attributes, we specify certain component networks for the former and the others for the latter. To let data tell which component network uses which attributes, we use a new exclusive group Lasso (least absolute shrinkage and selection operator) regularization method. It combines exclusive Lasso [89,90] and group Lasso [91] to reach 2 goals jointly. First, in each component network, every attribute competes with every other attribute. When one is employed, the others are less likely to be employed. Second, in each component network, all of the input vector weight matrix elements connecting to the same attribute tend to become nonzero (or zero) concurrently. Nonzero means the attribute is employed. We will use TensorFlow [62] to train MCLSTM and use our previous method [84,92] to automate hyperparameter value selection.

Kale et al [93-97] showed that in a deep neural network, we can use training instances that incur the highest activations of a neuron to find clinically meaningful features. After training the MCLSTM network, we proceed as follows to identify zero or more such features from every memory cell vector element at the final time step of the network. First, we find several training instances that incur the highest activations in the memory cell vector element. Second, in each of those training instances, we find one or more segments of the input vector sequence termed effective segments, each tending to represent a useful temporal feature. Third, we partition all spotted effective segments into multiple clusters and visualize each cluster separately to identify zero or more clinically meaningful temporal features. As shown in Wang et al [98], such a visualization could help us find and address data quality issues such as an implausible order of events, boosting model accuracy. For each identified feature, Dr Luo and a clinician in our team will jointly arrive at an exact mathematical definition of an extracted feature. Many extracted features capture trends more precisely than the raw features learned by LSTM. This also boosts model accuracy.

#### Aim 1’s Final Predictive Models

We will use the extracted temporal features to convert longitudinal data to tabular data, with 1 column per feature, and add static features. Health care system features such as the number of a physician’s patients of a given race can boost model accuracy [64,65,99] and are included as static features. We will employ Weka [61] to construct Aim 1’s final predictive models. As shown in Aim 4, these models are suitable for automatic explanation. We will use supervised algorithms and our previous method [84,92] to automatically select the algorithm, feature selection technique, imbalanced class handling method, and hyper-parameter values among all of the applicable ones. We will do manual fine-tuning if needed.

Using historical data up to the prediction time point, we will build 3 sets of models, one for each of 3 combinations: COPD at IH, KPSC, and UWM. For each of IH, KPSC, and UWM, the corresponding set of COPD models will be built for all of the COPD patients in that health care system. Unlike integrated health care systems IH and KPSC, UWM has most of its patients referred from other health care systems and has fairly incomplete data on many of its patients. To reduce incomplete data’s impact on model accuracy, we will use our previous constraint-based method [100] to find patients tending to receive most of their care at UWM and build models on and apply models to them. Previously, we showed that a good constraint for all types of UWM patients on average is that the patient lives within 5 miles of a UWM hospital and has a UWM primary care physician [100]. Yet, the optimal distance threshold could vary across various types of patients because of their different characteristics. Intuitively, a UWM COPD patient is likely to keep using and get a large portion of his/her care from UWM, even if the patient lives at some distance away from the closest UWM hospital. In comparison, a patient who visited a UWM emergency room once owing to a car accident may no longer use UWM after that visit. We will use the approach in our previous work [100] to find an optimal distance threshold for COPD patients. As noted earlier, we will develop and test our techniques on asthma as well.

#### Accuracy Evaluation and Justification of the Sample Size

The discussion below is for IH data. The cases with KPSC and UWM data are similar. As we need to compute outcomes for the subsequent year, we essentially possess 13 years of IH data over the past 14 years. We will train and test models in a usual way. We will do a stratified 10-fold cross-validation [61] on the data in the first 12 years to train models and to estimate their accuracy. The data in the 13th year will be employed to gauge the performance of the best models, mirroring future use in clinical practice. We will select the best model using the standard performance metric AUC [61]. A care management program typically enrolls 1% to 3% of COPD patients [32]. Of the upper 1% of COPD patients the model projects to be at the highest risk of using the hospital, we will report the percentage of patients using the hospital in the subsequent year. For a program taking 1% of COPD patients based on the model’s prediction results, this percentage reflects the degree of correct enrollment. To find the variables vital for high accuracy, we will conduct backward elimination [74] to remove features on the condition that accuracy does not drop >0.02. We will compare the variables vital for high accuracy on IH data with those on KPSC and UWM data. Using the variables available in both the IH and KPSC/UWM datasets, we will build the best predictive model on IH data and compare the model’s accuracy on IH data with that on KPSC/UWM data.

We will test the hypothesis that using our techniques can boost model accuracy. To do this, we will use a 2-sided Z test to compare the AUCs of 2 predictive models built in a way like that in Obuchowski [101]. The first predictive model will use the best machine learning algorithm and take all features essential for high accuracy. The second model will be adapted from those in the literature. For each predictive model for hospital usage reported in the literature [19-28], we will retrain it on our dataset using the attributes appearing in both the original model and our dataset. The most accurate one of the retrained models will be the second model. Our hypothesis is as follows:

1. Null hypothesis: The first model reaches the same AUC as the second.
2. Alternative hypothesis: The first model reaches a higher AUC than the second.

The categorical outcome variable of hospital usage has 2 possible values (classes). To the best of our knowledge, every predictive model for hospital usage reported in the literature reaches an AUC <0.8 [19-28]. “Using a two-sided Z-test at a significance level of 0.05 and assuming for both classes a correlation coefficient of 0.6 between the two models’ prediction results, a sample size of 137 instances per class has 90% power to detect a difference of 0.1 in AUC between the two models,” [65] like an increase of AUC from 0.8 to 0.9. The IH data in the 13th year include around 35,000 COPD patients, offering enough power to test our hypothesis. This conclusion remains valid if the actual correlation coefficient differs somewhat from the assumed one.

### Aim 2: Assess Using Temporal Features’ Impact on Risk Warning Timeliness

The discussion below is for IH data. The cases with KPSC and UWM data and with asthma are similar.

#### Outcome of the Number of Days of Early Warning the Model Provides for the Patient and the Estimation Approach

Consider a predictive model and a patient who used the hospital on date *D* in the 14th year. The outcome is the number of days of early warning the model provides for the patient. To measure the number, we find the first date *D'* (*D*-365≤ *D'* ≤ *D*-1) such that if we use *D'* as the prediction time point and input the patient’s history up to *D'* into the model, the model predicts hospital use in the subsequent year. In this case, the model warns the first hospital use *k* (0≤ *k* ≤ *D-D'*) days in advance, with *D'* + *k* being the first day between *D'* and *D* when the patient used the hospital. *k* is the outcome number. Otherwise, if the model still predicts no hospital use when we reach *D*-1, the model warns zero day in advance and zero is the outcome number. We expect using our techniques will raise the outcome number. We will assess the outcome on the cohort of COPD patients who ever used the hospital during the 14th year. For these patients, the average number of days of early warning given by the model shows how timely it warns.

#### Outcome Evaluation and Justification of the Sample Size

We will test the hypothesis that using our techniques can boost risk warning timeliness. To do this, for the patient cohort, we will use an *F* test to compare the number of days of early warnings provided by the 2 models mentioned in Aim 1’s accuracy evaluation section, assuming a Poisson model with an offset of 365 days. Our hypothesis is as follows:

1. Null hypothesis: The number of days of early warning provided by the first model is the same as that provided by the second.
2. Alternative hypothesis: The number of days of early warning provided by the first model is larger than that provided by the second.

Assuming the number of days of early warning has an exponential distribution and employing an *F* test at a one-sided significance level of 0.05, a sample size of 600 patients offers 80% power to detect a minimum raise of 27.8 days of early warning by the first model, when the second model warns, on average, 180 days in advance. About 2000 COPD patients ever used IH hospitals during the 14th year, giving enough power to test our hypothesis. The conclusion remains valid if the actual situation differs somewhat from the assumed one.

For Aims 1 and 2, our goal is to reach a boost of ≥0.1 in accuracy and ≥30 days in risk warning timeliness, respectively. If we cannot reach this goal on the entire COPD patient group, we will construct distinct models for differing patient subgroups. The patient subgroups are described by characteristics such as age or co-morbidity, which are often independent variables in the original predictive models. If we still cannot reach this goal, we will do subanalyses to find the patient subgroups, for which our predictive models show good performance, and then apply our ultimate predictive models only to these patient subgroups.

### Aim 3: Assess Using Temporal Features’ Potential Impact on Outcomes Via Simulations

To assess the value of a predictive model for future clinical deployment, we need to appraise care management outcomes if the model is adopted and decide how to generalize the predictive model to other sites gathering differing sets of variables. Our predictive models will be constructed on IH, KPSC, and UWM data. Our simulations will guide how to employ the predictive models in other health care systems. No previous study has decided the variables most crucial for COPD and asthma model generalization. We will apply our simulation method to care management of (1) COPD patients, (2) asthmatic children, and (3) asthmatic adults.

#### Outcomes of the Number of Inpatient Stays and the Number of Emergency Room Visits in the Subsequent Year and the Estimation Approach

The number of inpatient stays in the subsequent year is the primary outcome. The number of emergency room visits in the subsequent year is the secondary outcome. The following discussion focuses on IH data and inpatient stays. The cases with KPSC and UWM data and/or emergency room visits can be handled similarly. From statistics reported in the literature [102,103], we will obtain the percentage of inpatient stays, *p,* a care management program can help avoid. Given a set of variables, we will adopt the same method used in Aim 1 to train a predictive model on the data in the first 12 years. For the data in the 13th year, we will gather prediction results, then estimate the outcome. Consider a patient who will have *n_e_* inpatient stays in the subsequent year without enrolling in the program. If the patient gets enrolled, for each inpatient stay of the patient, we will simulate whether it will occur or not based on probability 1-*p*. The gross outcome estimate will be the sum of the estimated outcomes of all patients. Adopting a similar method, we will find the minimum accuracy required for the predictive model to be valuable in clinical practice.

#### Sensitivity Analysis

IH, KPSC, and UWM collect many variables. Another health care system may collect fewer. To ensure generalizability, we will evaluate various variable combinations and obtain the estimated outcomes when the revised model is adopted. These estimates will pinpoint crucial variables. If a crucial variable is not available in a given health care system, these estimates can hint alternative variables having minimal adverse impact on the outcomes.

We will employ a variable grouping approach relating variables likely to co-exist, such as those linked in a laboratory test panel, according to the judgment of our clinical experts. We will create and post a table showing many possible combinations of variables by groups, encompassing the trained parameters and the simulated outcomes of the predictive model. A health care system wanting to deploy the model can employ this table to estimate the expected outcomes in the system’s data environment, as well as to determine the variables to be gathered. The table has 3 columns, one for each of IH, KPSC, and UWM. Many variables collected by IH, KPSC, and UWM and used in this study are commonly available in many other systems. Thus, all variables used in each of many rows in the table will already exist in these systems.

#### Outcome Evaluation and Justification of the Sample Size

The following discussion focuses on IH data. The cases with KPSC and UWM data are similar. We will employ McNemar test to compare the paired-sample outcomes reached by the 2 predictive models mentioned in Aim 1’s accuracy evaluation section. We will test 2 hypotheses: using our techniques will link to a potential drop in (1) inpatient stays and (2) emergency room visits in the subsequent year. Our primary hypothesis is as follows:

1. Null hypothesis: The number of inpatient stays in the subsequent year reached by the first model is the same as that reached by the second.
2. Alternative hypothesis: The number of inpatient stays in the subsequent year reached by the first model is smaller than that reached by the second.

Among the patients truly at high risk for future hospital use, the first model will find some missed by the second and vice versa. Assuming the former cuts inpatient stays in the subsequent year by 5% and the latter increases them by 1%, at a one-sided significance level of 0.05, a sample size of 251 instances provides 80% power to verify the primary hypothesis. The IH data in the 13th year cover about 35,000 COPD patients, offering enough power to test the primary hypothesis.

### Aim 4: Automatically Identify Actionable Temporal Risk Factors for Each Patient at High Risk for Future Hospital Use and Assess the Impact on Prediction Accuracy and Outcomes

Care managers currently give finite input on the patient to the other clinical care team members. Owing to bandwidth constraints, care managers can afford to examine only a finite number of patients top ranked by the predictive model—those whose projected risk for future hospital use is over a given threshold like the 95th percentile. For those patients, we will automatically explain early warnings, identify actionable temporal risk factors, and suggest tailored interventions. This helps care managers make enrollment decisions and form tailored care plans. This also enables care managers to pass actionable information on to the other members in the clinical care teams and collaborate more closely with them. To implement the new function, we will improve our previous method [59] to automatically explain a machine learning model’s prediction results without incurring any accuracy loss. For nonlongitudinal tabular data, our previous method separates explanation and prediction by employing 2 models simultaneously, each for a distinct purpose. The first model gives predictions to maximize accuracy. The second employs class-based association rules mined from historical data to explain the first model’s results. Our previous method cannot handle longitudinal attributes and has not yet been applied to COPD, asthma, or care management.

As mentioned in Aim 1, we will use temporal features to convert longitudinal data to tabular data, with 1 column per feature. Then we can apply our previous automatic explanation method [59]. Each patient is represented by the same set of features and is marked as either high risk for future hospital use or not. From historical data, our method mines association rules linked to high risk. An example rule is as follows: the ratio of inhaled steroid to beta agonist dispensing to the patient decreased over 12 months AND sulfur dioxide level was ≥3 parts per million for ≥5 days in the past week ⟶ high risk. The first item on the left-hand side of the rule is an actionable temporal risk factor. Two interventions for the first item are to (1) assess COPD controller medication compliance and change, prescribe, or raise the dose of the medication if needed and (2) assess the patient for COPD triggers and ensure the patient stays away from them. Our paper [71] listed several interventions for a few other temporal risk factors. By discussion and consensus, the clinical experts in our team will check the mined rules and drop those making little or no sense clinically. For every rule that remains, our clinical team will mark the actionable temporal risk factors in it and list zero or more interventions that address the reason shown by the rule.

At the time of prediction, for every patient our most accurate model projects to be at high risk for future hospital use, we will find and show all of the association rules whose left-hand side conditions the patient satisfies, and list the interventions linking to these rules as our suggestions. Each rule shows a reason why high risk is anticipated for the patient. Users of our automatic explanation function can give feedback to help us find and drop unreasonable rules [64].

#### Boost Automatic Explanations’ Coverage of Patients, Model Accuracy, and Generalizability

For a nontrivial portion of patients, our previous automatic explanation method [59] cannot explain the prediction results of the model. Our previous method employs a conventional approach to mine association rules at a specific level of 2 parameters: minimum confidence and support. This approach is suboptimal for imbalanced data. There, the outcome variable takes the high-risk value for future hospital use much more often than the low-risk one. Adopting the same minimum support for both values is inadequate [104]. If the minimum support is too small, the rule mining process will form many overfitted rules, making it daunting for clinicians to check all of the mined rules. If the minimum support is large, we cannot identify enough rules for the high-risk value. Consequently, for many patients projected to be at high risk for future hospital use, we cannot explain the prediction results of the model.

To enlarge automatic explanations’ coverage of patients, we will use a new technique. It generalizes to many clinical applications and is sketched as follows, with more details given in our design paper [71]. We will use Paul et al’s [104] approach to mine association rules, by replacing support by value-specific support termed commonality. This has 2 advantages. First, the rule-mining process produces fewer overfitted rules, cutting the time clinicians need to check the mined rules. Second, we obtain more rules for the high-risk value of the outcome variable. Thus, for more patients projected to be at high risk for future hospital use, we can explain the prediction results of the model.

Using automatic explanations and the method described in our paper [64], we will find and drop uninformative features and retrain the predictive model. For the model, this can boost its accuracy, as well as make it generalize better to other health care systems beyond where it was originally built. On nonmedical data, Ribeiro et al [105] showed a similar method with a narrower scope boosted model accuracy by approximately 10%.

#### Performance Evaluation

We will compare the association rules obtained from IH, KPSC, and UWM data. The following discussion focuses on IH data. The cases with KPSC and UWM data are similar. We will do analyses similar to those in Aim 1 to compare using our new techniques in Aim 4 versus the current method of offering no explanation. We will compare the outcomes of the number of inpatient stays and the number of emergency room visits in the subsequent year and the accuracy reached by the 2 models: the best ones produced in Aims 1 and 4. We will test 3 hypotheses: using our new techniques in Aim 4 will link to a potential drop in (1) inpatient stays and (2) emergency room visits in the subsequent year and (3) boost prediction accuracy. Our primary hypothesis is as follows:

1. Null hypothesis: The number of inpatient stays in the subsequent year reached by the second model is the same as that reached by the first.
2. Alternative hypothesis: The number of inpatient stays in the subsequent year reached by the second model is smaller than that reached by the first.

We will employ McNemar test to compare the paired-sample outcomes reached by the 2 models.

Among the patients truly at high risk for future hospital use, the second model will find some cases missed by the first and vice versa. Assuming the former cuts inpatient stays in the subsequent year by 2.5% and the latter increases them by 0.5% and using McNemar test, at a one-sided significance level of 0.05, a sample size of 503 instances provides 80% power to verify the primary hypothesis. The IH data in the 13th year cover approximately 35,000 COPD patients, offering enough power to test the primary hypothesis.

To assess our technique’s impact on model generalizability, we will compare 2 predictive models’ accuracy on KPSC/UWM data. The first model is the best one produced in Aim 1 on IH data using the variables available in both the IH and KPSC/UWM datasets. The second is produced by using our technique to drop uninformative features from the first model and retrain it on IH data. We will develop and test our techniques on asthma as well.

### Aim 5: Assess Actionable Information’s Impact on Clinicians’ Acceptance of Early Warnings and on Perceived Care Plan Quality

As an essential preparatory step for future clinical deployment, we will evaluate actionable information’s impact on UWM care managers and physicians’ decision making in a test setting. For physicians, we will use primary care physicians, pulmonologists, and allergists managing COPD patients. The discussion below focuses on care managers. The case of evaluating with 10 physicians is similar.

#### Subject Recruitment

As an operational project at UWM, we are working on COPD outcome prediction and can access approximately 25 UWM care managers for adults. By making announcements in their email lists and personal contact, we will recruit 10 care managers. We will adopt purposeful sampling to ensure adequate variability in work experience [106]. All evaluation test participants will give consent and be up-to-date on privacy and information security policy training required by UWM. Participants will obtain pseudonyms, connecting their responses to questions to protect privacy. After completing the task, each will obtain US $2400 as compensation for participation for approximately 40 hours of work. We will conduct 2 experiments.

### Experiment 1

#### Procedures

From the IH data in the 13th year, we will randomly select 400 IH COPD patients who used the hospital in the subsequent year and automatically explain the prediction results of the best IH model built in Aim 4. We will use patients outside of UWM to help ensure no care manager is aware of any of those patients’ outcome in the subsequent year. We will show every care manager a different subset of 40 patients and proceed in 3 steps:

- Step 1: For every patient, we will present the historical de-identified patient attributes and the prediction result to the care manager and ask him/her to record the enrollment decision and interventions, if any, that he/she plans to use on the patient. For the historical patient attributes, we will show the static attributes’ values at the top, followed by the longitudinal attributes’ values in reverse chronological order. No care manager will see any automatic explanation in this step.
- Step 2: For every patient, we will present the automatic explanations and their linked interventions to the care manager and survey him/her using both semistructured and open-ended questions. The automatic explanations will appear as a list of association rules. Below each rule is the list of interventions linked to the rule. The questions will include whether these explanations would change the enrollment decision for the patient, whether he/she believes they would improve care plan quality, their usefulness on a 1 to 7 scale with anchors of not at all/very useful, and their perceived trustworthiness on a 1 to 7 scale with anchors of not at all/completely. Our questionnaire will embrace a text field for writing comments.
- Step 3: We will use the standard Technology Acceptance Model (TAM) satisfaction questionnaire [107] to survey the care manager about the automatic explanations. A technology is unimportant unless people will accept and use it. Developed based on multiple well-accepted behavioral theories, TAM is the most widely adopted model of people’s acceptance and usage of a technology. The TAM satisfaction questionnaire will measure the perceived usefulness and the perceived ease of use of automatic explanations. Perceived usefulness is known to link strongly to future usage intentions and to actual function usage [108,109]. Multiple studies have demonstrated the validity and reliability of the TAM satisfaction questionnaire [110,111].

#### Analysis

We will adopt the inductive approach described in Patton et al [106,112] to conduct qualitative analysis. Care managers’ textual comments will be put into ATLAS.ti qualitative analysis software [113]. In total, 3 people in our research team will independently highlight quotations on prediction results and automatic explanations for all records. Quotations will be examined, classified into precodes, and merged into categories via discussion and negotiated consensus in several iterations. We will find general themes via synthesis of categories. The quantitative analyses will include giving descriptive statistics for every quantitative outcome measure. We will test the hypothesis that for the patients who will use the hospital in the next year, giving actionable information will improve the perceived care plan quality. Our hypothesis is as follows:

1. Null hypothesis: For the patients who will use the hospital in the next year, the care manager does not believe that showing the automatic explanations and their linked interventions would improve care plan quality.
2. Alternative hypothesis: For the patients who will use the hospital in the next year, the care manager believes that showing the automatic explanations and their linked interventions would improve care plan quality

We will fit a random effect logistic model to account for correlation among the outcomes of the same care manager on whether the perceived care plan quality is improved.

#### Justification of the Sample Size

Assuming a moderate intra-class correlation of 0.1 within the same care manager on the outcome of whether the perceived care plan quality is improved, a sample size of 40 instances per care manager for 10 care managers is equivalent to totally 82 independent instances after adjusting for the clustering effect. At a 2-sided significance level of 0.05, we will have 80% power to identify a 9.7% increase in the odds of improving the perceived care plan quality with actionable information. A similar conclusion holds if the actual correlation differs somewhat from the assumed one.

If giving actionable information has no significant impact on the perceived care plan quality on the whole group of COPD patients, we will do subanalyses to find those patient subgroups on which significant impact occurs.

### Experiment 2

#### Procedures

We will randomly partition the 10 care managers into 2 disjoint groups: the intervention group and the control group. Each group has 5 care managers. From the IH data in the 13th year, we will randomly select 200 IH COPD patients who used the hospital in the subsequent year and whose data are unused in Experiment 1 and automatically explain the prediction results of the best IH model built in Aim 4. For each group, we will show every care manager in the group a different subset of 40 patients. With random assignment, each patient is shown to 2 care managers, one in the intervention group and the other in the control group. In the control group, for every patient, we will show the care manager the historical de-identified patient attributes and the prediction result but no automatic explanation. In the intervention group, for every patient, we will show the care manager the historical de-identified patient attributes, the prediction result, the automatic explanations, and their linked interventions. In both groups, we will ask the care managers to record their enrollment decisions.

#### Analysis

We will test the hypothesis that for a patient who will use the hospital in the next year, giving actionable information will increase the likelihood that a care manager decides to enroll the patient in care management. Our hypothesis is as follows:

1. Null hypothesis: For a patient who will use the hospital in the next year, the likelihood that a care manager in the intervention group decides to enroll the patient in care management is the same as that in the control group.
2. Alternative hypothesis: For a patient who will use the hospital in the next year, the likelihood that a care manager in the intervention group decides to enroll the patient in care management is higher than that in the control group.

We will fit a random effect logistic model to compare care managers’ enrollment decision outcomes between the intervention group and the control group, while accounting for correlation among the enrollment decision outcomes of the same care manager and correlation between the enrollment decision outcomes of the same patient examined by 2 care managers.

#### Justification of the Sample Size

Assuming a moderate intra-class correlation of 0.1 within the same care manager and within the same patient examined by 2 care managers on the enrollment decision outcome, a sample size of 40 instances per care manager for 10 care managers is equivalent to totally 74 independent instances after adjusting for the clustering effect. At a 2-sided significance level of 0.05, we will have 80% power to identify a 9.7% boost in the intervention group in the odds that a care manager decides to enroll the patient in care management. A similar conclusion holds if the actual correlations differ somewhat from the assumed ones.

As mentioned right before Aim 1, the above discussion focuses on COPD. Whenever we mention COPD, the same applies to asthma and will be developed and tested on asthma also in Aims 1 to 5.

### Ethics Approval

We have obtained from IH, UWM, and KPSC institutional review board approvals for this study.

## Results

We are currently downloading clinical and administrative data from the EDWs of UWM, KPSC, and IH. We plan to start data analysis in 2020 and finish our study in 2025.

## Discussion

### Clinical Use of Our Results

Care managers collaborate with the other members in the clinical care teams. We will automatically explain early warnings and suggest possible interventions to help clinical care teams form tailored care plans on the grounds of objective data. This could facilitate clinicians to review structured data in patient charts faster and enable closer collaboration between care managers and the other members in the clinical care teams. Once our methods find patients at the largest projected risks for future hospital use and provide explanations, clinicians will check patient charts, examine factors such as social dimensions and potential for improvement [102], and make care management enrollment and intervention decisions.

As time goes by, both the feature patterns linked to high risk for future hospital use and patient status keep changing. In clinical practice, we can re-apply our techniques regularly to the latest clinical, administrative, air quality, and weather data sets to move patients into and out of care management and to find new feature patterns over time.

As in the case with LSTM, with information from only one visit, our proposed predictive models can start making projections on the patient. Yet, all else being equal, we would expect the prediction accuracy and risk warning timeliness reached by our models to improve as the length of patient history increases.

### Generalizability

We will semiautomatically extract predictive and clinically meaningful temporal features from longitudinal data, solving an open computer science challenge [60]. Both our feature extraction and automatic explanation methods will help drop uninformative variables, reducing the variables used in the model. This boosts model generalizability and partly addresses the limitation that one study cannot afford to test models on all US patients. As Gupta et al [114] showed, many extracted features represent general properties of the attributes used in the features and can be valuable for other predictive modeling tasks. Using the extracted features to build a temporal feature library to aid feature reuse, we can cut down the effort required to construct models for other predictive modeling tasks.

The principles of our techniques are general, depending on no unique characteristic of a specific disease, patient cohort, or health care system. Care management is also widely used for patients with diabetes and heart diseases [34], where our techniques could be used. Our simulation will find out how to generalize a predictive model to other sites gathering differing sets of variables and those variables most crucial for generalization. We will use data from 3 health care systems IH, KPSC, and UWM to illustrate our techniques on the cases of COPD and asthma patients. These health care systems include 2 integrated systems (IH and KPSC), an academic system with most patients referred from other systems (UWM), and many heterogeneous facilities. These facilities cover 41 hospitals and 424 clinics spread over 3 large geographic areas, ranging from rural and community urban clinics staffed by a variety of clinicians including physicians, nurses, therapists, and advanced practice practitioners with limited resources to metropolitan tertiary care hospitals staffed by subspecialists. These systems use 4 different electronic health record systems: IH uses Cerner, HELP, and HELP2; UWM uses Cerner and Epic; KPSC uses Epic. Variation in scope of services, staff composition, geographic location, cultural background, patient population, health care system type, and electronic health record system allows us to find factors generalizable to other facilities nationwide. Our models will be based on the OMOP common data model [72] and its related standardized terminologies [73], which standardize clinical and administrative variables from ≥10 large US health care systems [115,116]. At a minimum, our models will apply to those systems using OMOP.

After extension, our techniques can be applied to various decision support applications and diseases and advance clinical machine learning: (1) more precise models giving earlier warnings will boost decision support tools for managing limited resources, such as planning for health care resource allocation [117] and automatically finding patients tending to be readmitted soon, triggering home visits by nurses to cut readmissions and (2) using our techniques can boost prediction accuracy and risk warning timeliness of other outcomes such as missed appointments [118], patient satisfaction [119], and adherence to treatment [120]. This will help target resources, such as reminder phone calls to cut missed appointments [118], or interventions to boost adherence to treatment [120].

We expect our more accurate predictive models giving earlier warnings to have value for clinical practice. Future studies will test our techniques on some other patient cohorts and diseases, implement our techniques at UWM, IH, and KPSC for care management for COPD and asthma, and evaluate the impacts in randomized controlled trials.

In summary, the techniques that will be developed in this study will advance machine learning for many clinical applications and help transform preventive care to be more efficient, effective, and timely. This will boost outcomes and save resources.

## Abbreviations

AUC: area under the receiver operating characteristic curve
COPD: chronic obstructive pulmonary disease
EDW: enterprise data warehouse
ICD-10: International Classification of Diseases, Tenth Revision
ICD-9: International Classification of Diseases, Ninth Revision
IH: Intermountain Healthcare
KPSC: Kaiser Permanente Southern California
Lasso: least absolute shrinkage and selection operator
LSTM: long short-term memory
MCLSTM: multi-component LSTM
OMOP: Observational Medical Outcomes Partnership
TAM: Technology Acceptance Model
UWM: University of Washington Medicine
